# Temporal discounting in major depressive disorder

**DOI:** 10.1017/S0033291713002584

**Published:** 2013-11-01

**Authors:** E. Pulcu, P. D. Trotter, E. J. Thomas, M. McFarquhar, G. Juhasz, B. J. Sahakian, J. F. W. Deakin, R. Zahn, I. M. Anderson, R. Elliott

**Affiliations:** 1 School of Medicine, Neuroscience and Psychiatry Unit, University of Manchester and Manchester Academic Health Science Centre, Manchester, UK; 2 Department of Pharmacodynamics, Faculty of Pharmacy, Semmelweis University, Budapest, Hungary; 3 Department of Psychiatry, University of Cambridge, and MRC Wellcome Trust Behavioural and Clinical Neuroscience Institute, Cambridge, UK; 4 School of Psychological Sciences, Neuroscience and Aphasia Research Unit, University of Manchester and Manchester Academic Health Science Centre, Manchester, UK

**Keywords:** Delay discounting, hopelessness, major depressive disorder, reward processing

## Abstract

**Background:**

Major depressive disorder (MDD) is associated with abnormalities in financial reward processing. Previous research suggests that patients with MDD show reduced sensitivity to frequency of financial rewards. However, there is a lack of conclusive evidence from studies investigating the evaluation of financial rewards over time, an important aspect of reward processing that influences the way people plan long-term investments. Beck's cognitive model posits that patients with MDD hold a negative view of the future that may influence the amount of resources patients are willing to invest into their future selves.

**Method:**

We administered a delay discounting task to 82 participants: 29 healthy controls, 29 unmedicated participants with fully remitted MDD (rMDD) and 24 participants with current MDD (11 on medication).

**Results:**

Patients with current MDD, relative to remitted patients and healthy subjects, discounted large-sized future rewards at a significantly higher rate and were insensitive to changes in reward size from medium to large. There was a main effect of clinical group on discounting rates for large-sized rewards, and discounting rates for large-sized rewards correlated with severity of depressive symptoms, particularly hopelessness.

**Conclusions:**

Higher discounting of delayed rewards in MDD seems to be state dependent and may be a reflection of depressive symptoms, specifically hopelessness. Discounting distant rewards at a higher rate means that patients are more likely to choose immediate financial options. Such impairments related to long-term investment planning may be important for understanding value-based decision making in MDD, and contribute to ongoing functional impairment.

## Introduction

Major depressive disorder (MDD) has been associated with reward processing impairments. Abnormal reward processing may also play a role in impaired occupational functioning, which has been identified as a crucial factor in the high economic costs of the disorder (Beddington *et al.* 2008). Hopelessness, a bleak outlook into the future and suicidal ideation are among symptoms of MDD (APA, 2000), and these symptoms may interfere with decisions requiring long-term economical investment planning. Temporal (delay) discounting may serve as an effective experimental probe of this behaviour. Tesch & Sanfey (2008) defined delay discounting as a fundamental dimension of financial decision making by which people choose between short-term gain maximization and long-term equity, depending on subjective valuation of money over time. They suggested that one of the key factors in determining trends in these financial decisions is individuals' preferences for having immediate rewards and delayed costs.

Previous reward processing research mostly made use of signal detection paradigms and investigated the relationship between monetary reward processing and anhedonia (inability to gain pleasure from activities that were previously enjoyed), which is one of the core symptoms of MDD (APA, 2000; Pizzagalli *et al.* 2005). These studies showed impaired response biases to monetary rewards in dysphoric individuals (Henriques *et al.* 1994; Juhasz *et al.* 2009), in individuals undergoing an experimental stress induction (Bogdan & Pizzagalli, 2006), and in people fulfilling clinical criteria for MDD (Pizzagalli *et al.* 2008). Time is an important variable in reward processing models. Signal detection paradigms manipulate the probability and frequency of winning monetary rewards, while keeping the reward size fixed across different conditions, but cannot address the impact of MDD on subjective valuation of different magnitudes of monetary rewards over time (i.e. delay discounting behaviour).

Delay discounting behaviour is typically assessed using monetary choice tasks that have been most frequently used to assess impulsive tendencies in people with various addictions (Kirby *et al.* 1999; Kirby & Petry, 2004; Bornovalova *et al.* 2005; Lawyer, 2008). However, discounting behaviour tends to correlate poorly with self-rated impulsivity on established personality scales in non-addicted populations (McLeish & Oxoby, 2007) and even seems limited to certain subtypes of impulsive behaviour in individuals with heroin addiction (Kirby *et al.* 1999).

From an economical perspective, delay discounting can also be used simply to define the degree to which individuals prefer short-term over long-time economical strategies (Read & Read, 2004). Previous discounting studies suggested that these individual preferences are influenced by both biological and environmental factors. In healthy subjects, discounting rates change over the lifespan, which may reflect neuroanatomical changes and/or changes in environmental factors (Read & Read, 2004; Whelan & McHugh, 2009). Environmental uncertainty imposed by external conditions also influences discounting rates. Individuals tend to prefer short-term rewards when they are traumatized by environmental conditions, such as the Wenchuan earthquake (Li *et al.* 2012), or financial deprivation (Chao *et al.* 2009). Under such conditions, where there is considerable uncertainty about the future, steeper discounting may be driven by a realistic evaluation of one's life circumstances rather than impulsivity.

As stated previously, MDD is characterized by anhedonia and hopelessness about the future as well as the present (Beck, 2005). It is possible that, because MDD is associated with a bleak future outlook and hopelessness, individuals may be expected to shift towards short-term economical decision-making strategies (i.e. higher discounting rates). We predict that these depressive symptoms will exert a significant effect particularly in evaluating rewards that are presented with the furthest delays. Existing studies have explored this hypothesis in mixed populations with bipolar and also with unipolar depression (Takahashi *et al.* 2008), showing lower discounting rates for the distant future but higher discounting rates for the near future in patients. A study in patients with late-life depression (Dombrovski *et al.* 2011) showed that lower discounting rates for delayed rewards are associated with high-lethality suicide attempts whereas low-lethality suicide attempters had higher discounting rates relative to both non-suicidal patients and healthy subjects. Healthy individuals with higher self-reported anhedonia (Lempert & Pizzagalli, 2010) or with experimentally reduced serotonin (one of the key neurotransmitters involved in MDD) (Schweighofer *et al.* 2008) have lower discounting rates for delayed rewards. Whether short-term experimental manipulations of serotonin is a good model for depression phenomenology is always debatable; in the context of the long-term projections involved in temporal discounting, the model may be particularly unsuitable. The two previous clinical studies (Takahashi *et al.* 2008; Dombrovski *et al.* 2011) showed a complex pattern of temporal discounting in depression dependent on both delay and patient characteristics. Therefore, we consider that temporal discounting warrants further investigation in a population of young to middle-aged adults with unipolar depression (current and remitted).

From the behavioural–economical perspective we presented earlier, we predicted that patients with current MDD would have higher discounting rates for delayed rewards (i.e. choosing the immediate reward option), particularly influenced by hopelessness about the future, which would make them less likely to invest into their future selves. The study of Dombrovski *et al.* (2011) showed that non-suicidal depressed individuals and suicidal ideators, and also low-lethality suicide attempters, tended to have higher discounting rates than non-depressed controls, and therefore it seems reasonable to predict higher discounting rates in our cohort.

We therefore sought to investigate delay discounting in patients with current unipolar MDD. Furthermore, we investigated whether delay discounting abnormalities may represent a state feature of depression. Recent evidence suggests that even patients with remitted MDD (rMDD) may show some abnormalities in emotional and reward processing (Eshel & Roiser, 2010; Green *et al.* 2013). However, if (as hypothesized) impaired discounting reflects symptoms, particularly hopelessness, abnormalities in MDD should normalize as symptoms remit. We therefore recruited patients with current and remitted MDD and healthy subjects, to test the predictions that patients with MDD will have a higher discounting rate for future rewards in long delays and that this behavioural tendency will not be seen in a group with fully remitted symptoms.

## Method

### Participants

The study obtained ethical approval from the North West/Manchester South National Health Service (NHS) Research Ethics Committee. Participants were recruited using online and print advertisements. Initial suitability was assessed with a telephone pre-screening interview and the use of an online survey. Written informed consent was obtained from all participants.

#### Inclusion/exclusion criteria

Patients with MDD fulfilled criteria for a current major depressive episode (MDE) according to DSM-IV-TR (APA, 2000). The clinical interviews were conducted by trained researchers. We excluded people with current or history of substance use disorders, psychotic disorders, clinically significant levels of suicide risk [in the acute phase of a previous attempt and scores ⩾5 on the Montgomery–Asberg Depression Rating Scale (MADRS) item 10], bipolar depression, and any other Axis I anxiety disorders as the likely cause of the current MDE and any other neurological disorders in the MDD group. Participants in the rMDD group fulfilled criteria for a past DSM-IV-TR MDE. Exclusion criteria for the rMDD group were similar but included currently meeting diagnostic criteria for MDD or taking psychotropic medication. The healthy control group had no current or past Axis I disorders.

In total, 29 healthy control participants, 29 individuals with rMDD and 24 patients with current MDD (11 with medication; see online Supplementary Tables S1 and S2 for information on clinical groups) were included in the final analysis. One patient (male, aged 43 years, non-medicated) with current MDD was excluded on the basis of current hypomanic symptoms that were not present at the time of the telephone screening interview.

### Materials and procedures

#### Clinical interview procedure

Participants were invited for a clinical interview in which trained researchers (E.P., P.D.T. and E.J.T.) conducted the Mood Disorders Module A and the psychotic screening of the Structured Clinical Interview for DSM-IV-TR (SCID; First *et al.* 2002). The Mini International Neuropsychiatric Interview (MINI; Sheehan *et al.* 1998) was conducted with all the participants and relevant SCID modules were used to make a full assessment. The MADRS, the Global Assessment of Functioning (GAF) scale (Axis V, DSM-IV) and the Social and Occupational Functionality Assessment Scale (SOFAS; only for patients with MDD) (Axis V, DSM-IV) were used.

#### The monetary choice task

The monetary choice task was based on Kirby *et al*. (1999) and contained 27 items asking participants to choose between two monetary offers: one available today and a larger one available at a delay (see Table 1 for examples). In this task, the delays varied between 7 and 186 days and rewards varied between £11 and £85 (the range comprising all immediate and delayed rewards). We followed Kirby *et al.*'s classification of rewards into three categories (small, medium and large) but converted the original task into UK currency (GBP; £). Following the methodology of Kirby *et al.* (1999) for delayed rewards (always larger than immediate rewards), small rewards were from £25 to £35, medium rewards were from £50 to £60, and large rewards were from £75 to £85. The immediate rewards varied between £11 and £80, always being smaller in magnitude than the delayed reward size in each temporal discounting proposal. We used a computerized version of this task. The monetary choices were presented in the same order as they were presented in Kirby *et al*. (1999). Before starting the task, participants were asked to read the instructions on the computer screen and any questions were clarified. Participants completed this task in a quiet room designated for testing purposes. The participants' choices on the task did not affect the amount of reimbursement they received for participation; we used a hypothetical version of the task.

**Table 1.** Examples of monetary choices in the delay discounting task^a^ (seven out of 27 time points)

Discount rate ranking	Reward today in GBP (*V*)	Future reward in GBP (*A*)	Delay in days (*D*)	Hyperbolic discount value when indifferent [equation (2)]	Hyperbolic discount rate (*k*) when indifferent [equation (1)]
1	11	30	7	0.090476	0.246753
2	15	35	13	0.043956	0.102564
3	27	50	21	0.021905	0.040564
4	40	55	62	0.004399	0.006048
5	49	60	89	0.002060	0.002522
6	67	75	119	0.000896	0.001003
7	78	80	162	0.000154	0.000158

a The selection was made so that both the delay in days and the future reward were sorted from the minimum to the maximum value. Kirby *et al*. (1999) suggested using equation (1).

### Data analysis

The *k* coefficient, which designates the discounting rate for delayed reward at any indifference point (i.e. the point in which participants do not discriminate between immediate and delayed rewards within any reward size category), was calculated using equation (1):
(1)
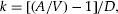
where *A* is the amount of the delayed reward, *V* is the subjective value of the delayed reward and *D* is the length of delay. Established indifference points for different rewards are plotted on a graph to establish a discounting curve for any individual. Previous research shows that hyperbolic equations explain the discounting data better than alternative exponential equations that make the assumption that the rate of discounting remains constant over time (Mazur, 1987; Murphy *et al.* 2001; Myerson *et al.* 2001; Green & Myerson, 2004). The hyperbolic discount function is obtained as:
(2)
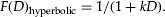
Individual discounting coefficients (*k*) for small, medium and large rewards, along with their geometric means as a separate score, were computed in Microsoft Office Excel. Descriptive and between-group analyses were conducted with SPSS version 20.0 (SPSS Inc., USA). We used a general linear model (GLM) to explore any interaction between clinical status and temporal discounting at different rewards sizes. To investigate the appropriateness of this model for the data, we undertook investigations of the model assumptions using residual plots (Neter *et al.* 1996, pp. 778–781). Both the normal quantile–quantile plot of the model residuals and the plot of the residuals against the fitted values of the model indicated that the errors were not satisfactorily normally distributed, and that the variance was not equal across all groups. To successfully transform the data so that these assumptions were better met, we used the Box–Cox procedure (Sakia, 1992) to search for a possible power transform. The Box–Cox procedure suggested a maximum likelihood estimate of *λ* = 0, and as such a natural log transform was performed on the outcome vector. Refitting the model on the transformed data and inspecting the residual plots suggested that the model assumptions were now satisfactorily met. We also used a one-way ANOVA to compare discounting behaviour between our groups. In line with our specific hypothesis, we undertook a simple main effects *post-hoc* investigation of the significant delayed reward size × group interaction term from the full model for large-sized rewards only (which were presented with the furthest mean delays). We fit a one-way ANOVA model using diagnostic group as the single between-subjects factor. The simple main effects *F* ratio was computed using the estimated mean square for the main effect of group from the one-way model as the numerator, and the mean square of the error from the original full GLM as the denominator (as the best unbiased estimator of the residual error; Langsrud, 2003). The *p* value was then computed from the upper tail of the null *F* distribution with df = 2,79. We used the Tukey–Kramer pairwise comparison procedure for unequal group sizes (Hayter, 1984) on significant differences. We also investigated the relationship between continuous clinical measures and discounting rates by means of conducting correlational analyses and used Bonferroni correction when reporting significant correlations. The reward magnitude and reward delay correlated significantly (*r* = 0.533, *p* = 0.004) in the monetary choice task (i.e. larger rewards were associated with longer delays). To maintain consistency with the rest of the literature using the same paradigm, we present the results with respect to reward magnitude (i.e. large-sized rewards) instead of reward delay.

#### Discount rate estimation procedure

Using the methodology suggested by Kirby *et al*. (1999), a discounting coefficient for each of the monetary choices was established by using equation (1). Calculation of an individual composite discounting coefficient for any given reward size uses ‘indifference points’ at which participants cannot choose between two monetary choices. For example, in a question that asks participants to choose between ‘£14 today’ and ‘£25 in 19 days’, a participant with a discounting rate higher than 0.041 would choose the immediate monetary option. If the same participant chooses the reward at a delay when they are asked to choose between ‘£15 today’ and ‘£35 in 13 days’, they would have a discounting rate less than 0.10. The composite discounting coefficient for this participant for small-sized rewards would be somewhere between these two anchoring points and, following the recommendations of Kirby *et al*. (1999), is calculated by taking the geometric mean of these two indifference points, therefore it would be 0.064.

When participants' choices were not consistent within a single value of the discounting coefficient, inconsistencies were resolved by taking the geometric mean of all the indifference points within the streak of inconsistent responses, as suggested by Kirby *et al*. (1999).

## Results

### Participants and demographics

Table 2 displays basic demographic and clinical information for all participants. The groups did not differ significantly in age and years of education but the current MDD group had a significantly higher number of males. Healthy subjects and people with rMDD had MADRS scores that were well below the cut-off for depression (<10) (Hawley *et al.* 2002), but the remitted MDD group showed slightly higher scores than controls. Both of these groups had GAF scores indicating minimal or absent symptoms (>80). Patients with current MDD had significantly higher MADRS and lower GAF scores. All of the patients with current MDD reported clinically significant levels of anhedonia based on the SCID assessment. Hopelessness scores were based on the ninth item of the MADRS. It has been suggested that this methodology is a clinically valid approach in measuring the severity of individual depressive symptoms (Desseilles *et al.* 2012). Currently depressed patients had significantly higher hopelessness scores (see Table 2 for further results).

**Table 2.** Group comparison on demographic and basic clinical variables

	Control (*n* = 29)	Remitted MDD (*n* = 29)	Current MDD (*n* = 24)	Test statistic	*p* value
Age (years)	38 ± 6.6	38.34 ± 5.9	38.25 ± 10.5	0.015^a^	0.985
Education (years)	17.3 ± 2.8	17 ± 3.1	16.2 ± 3.5	0.901^a^	0.411
Gender, female	19	23	11	6.61^b^	0.037*
MADRS	2 ± 2.7	3.9 ± 3.4	33 ± 4.3	52.193^c^	0.000*
MADRS-9	0.27 ± 0.58	0.39 ± 0.58	3.75 ± 0.94	57.715^c^	0.000*
GAF	90 ± 5.6	87.1 ± 5.1	58.7 ± 8.7	54.521^c^	0.000*
SOFAS	–	–	57.1 ± 10.0	–	–

MDD, Major depressive disorder; MADRS, Montgomery–Asberg Depression Rating Scale (MADRS-9 refers to hopelessness scores); GAF, Global Assessment of Functioning; SOFAS, Social and Occupational Functionality Assessment Scale.

Values are given as number or mean ± standard deviation.

a One-way ANOVA (df = 2,79).

b Pearson's *χ*^2^ (df = 2).

c *χ*^2^ value in the Kruskal–Wallis test (df = 2, showing asymptomatic significance).

* Significant at *p* ⩽ 0.05 threshold, two-tailed.

Patients with MDD had a significantly higher number of males in the population, significantly higher overall MADRS scores and hopelessness scores, and significantly lower GAF scores compared with the rest of the groups. Remitted MDD and controls do not differ on any of the affective measures.

### Discounting rates

We analysed the discounting task by fitting a GLM in which delayed reward size (small, medium, large) was treated as a within-subjects factor and diagnostic group (control, rMDD, current MDD) was treated as a between-subjects factor.

The results from the refitted log-transformed model suggest that there was a significant reward size × diagnostic group interaction (*F*_4,158_ = 3.968, *p* = 0.004) with a significant Type III main effect of delayed reward size (*F*_2,158_ = 53.146, *p* < 0.001) but no significant Type III main effect of diagnostic group (*F*_2,9_ = 1.230, *p* = 0.298). The results of the simple main effects analysis of the large rewards condition indicated a significant main effect of group (*F*_2,79_ = 8.955, *p <* 0.01). *Post-hoc* pairwise comparisons using the Tukey–Kramer procedure with Studentized range critical values (*q* < 0.01 for df_2,79_ = 4.24) suggested that patients with MDD have significantly higher discounting rates relative to healthy subjects and remitted patients [absolute difference>critical range: MDD>CON = 0.71>0.53; MDD>rMDD = 0.57>0.53; both significant at *p <* 0.01; healthy subjects and the remitted group were not significantly different (0.14<0.50)].

**Fig. 1.**
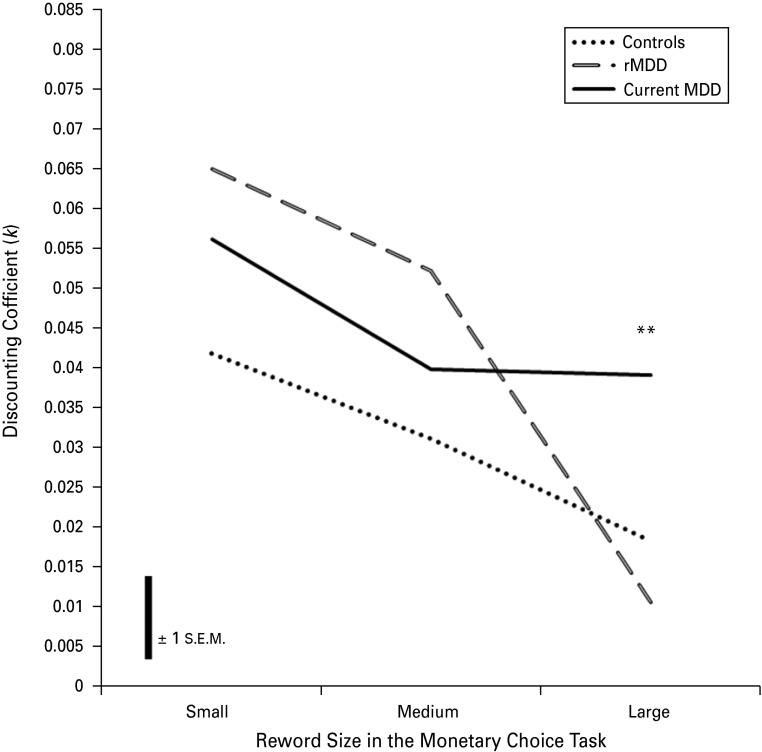
Graph showing mean discounting coefficients (*k*) against monetary reward size using the raw data before natural log transformation. Patients with major depressive disorder (MDD) had significantly higher discounting rates for large-sized rewards relative to healthy subjects and remitted patients with fully remitted MDD (rMDD) (** *p* < 0.01 for both comparisons). The scale bar shows ± 1 mean standard error (s.e.m.) across all reward sizes ( = 0.01); the s.e.m. for large-sized rewards is 0.008 (Control: 0.006, rMDD: 0.002, Current MDD: 0.015). The mean reward sizes for small-, medium- and large-sized rewards are £30, £55 and £80, respectively.

Finally, we computed one-sample *t* tests separately in each group using a triangular area under the curve (AUC) measurement [equation (3)] to investigate whether the change in discounting rates from medium- to large-sized rewards were significant. The AUC analysis helped us to confirm that clinical group × reward size interaction is influenced by abnormal temporal discounting in current depression, whereby depression selectively affects evaluation of medium- (*M*) to large-sized (*L*) rewards over time.
(3)
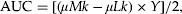
where *Y* is a constant based on the difference between mean large-sized rewards (£80) and mean medium-sized rewards (£55); therefore 25. We used the mean discounting rate for large-sized rewards (*μLk*) of each group as the test value in their respective one-sample *t* tests. The results showed that, in healthy subjects (df = 28, *t* = 2.178, *p* < 0.05) and remitted patients (df = 28, *t* = 3.957, *p* < 0.001), the change from medium- to large-sized rewards is significant whereas in patients with current MDD, the change is non-significant (df = 23, *t* = −1.030, *p* = 0.314). The groups did not differ significantly in the amount of change in discounting rates from medium- to large-sized rewards (*F*_2,79_ = 1.569, *p* = 0.215). Taken together, these analyses suggest that remitted patients and healthy subjects display comparable temporal discounting behaviour, whereas patients with current depression have significantly higher discounting rates for large-sized rewards, which is mainly influenced by the inability to evaluate medium- to large-sized rewards differently over time, resulting in a plateau of the discounting curve (Fig. 1).

### *Post-hoc* correlation analyses

We investigated the relationship between depressive symptoms, particularly hopelessness, and discounting rates for large-sized rewards. Exploratory analyses comprising all participants revealed that there was a significant correlation between MADRS and GAF scores (Spearman's *r*_s_ = −0.777, *p* < 0.001), and in the pooled MDD sample (comprising patients with current and remitted MDD; *n* = 53) we observed this relationship more strongly (Spearman's *r*_s_ = −0.855, *p* < 0.001). Our specific hypotheses concerned correlations between discounting behaviour and (i) GAF scores and (ii) hopelessness scores. As both of these scores correlated with depression severity (MADRS), we controlled for MADRS scores in these analyses. In the pooled MDD sample, GAF scores (as an indicator of general psychosocial functioning impairment) and the discounting coefficient for large-sized rewards showed a significant relationship (Pearson's *r* = −0.308, *p* < 0.01), controlling for MADRS scores. Furthermore, in the pooled MDD sample, discounting scores correlated significantly with hopelessness scores (Pearson's *r* = 0.394, *p* < 0.01), again controlling for MADRS; all correlations survived Bonferroni correction.

Finally, to control whether our findings were driven by medication effects or the gender distribution in the MDD group, we compared patients with and without medication and male patients with female patients. There were no significant differences for any of the delayed reward sizes within the MDD group between medicated and medication-free patients (*t* = −0.787, df = 22, *p* = 0.440), and between male and female subjects (*t* = 0.051, df = 22, *p* = 0.960).

## Discussion

The results of our study suggest that financial decision making in patients with MDD is associated with shorter-term financial reward preferences indicated by higher discounting rates for large-sized rewards relative to healthy subjects and remitted patients. We found that differences in discounting rates across reward sizes were modulated by clinical groups, such that MDD patients, relative to both control and rMDD groups, did not show a decrease in discounting rates between medium and large rewards. Higher discounting rates for large-sized rewards seem to be associated with lower scores on a measure of general psychosocial functioning (i.e. GAF) even when controlling for depression severity (i.e. MADRS scores). Furthermore, we have shown that discounting rates for future rewards correlated significantly with the severity of hopelessness in the depression group. Finally, we found that patients with fully remitted symptoms did not differ significantly from healthy subjects in terms of temporal discounting behaviour.

As expected, we have shown a significant clinical group by delayed reward size interaction, with no significant main effect of clinical diagnoses, consistent across all delayed reward sizes. One-way ANOVA confirmed our *a priori* hypothesis that our groups would be different in delay discounting coefficients for large-sized rewards. *Post-hoc* pairwise comparisons revealed that patients with current MDD had significantly higher discounting rates relative to both healthy subjects and remitted patients. Significant correlations between severity of hopelessness in the joint MDD group and the discounting coefficient for large-sized rewards supported our prediction based on Beck's cognitive triad (Beck, 2005).

Previous studies have argued that individuals with self-reported anhedonia demonstrated farsighted decisions because present anhedonia blunts responses to immediate rewards and these individuals would imagine themselves enjoying monetary rewards more in the distant future than in the present time (Lempert & Pizzagalli, 2010). However, it is questionable whether self-reported anhedonia in healthy subjects is a reliable model for MDD. Beck's cognitive triad model argues that MDD is characterized by a negative view of the future as well as the present. In a forced choice paradigm, it may be that pessimism about the future is a stronger influence on behaviour than present anhedonia. Remission of future pessimism and hopelessness may explain the absence of significant differences between the remitted group and healthy subjects for large-sized rewards.

The present findings advance our understanding of impairments in MDD associated with reward processing. Previous studies mainly considered impairments contingent upon frequency and probability of winning financial rewards, but not how patients with MDD subjectively evaluated their magnitudes over time (Henriques *et al.* 1994; Pizzagalli *et al.* 2005, 2008). In the current study we have shown that patients with MDD were insensitive to the changes in the magnitude of medium- to large-sized financial rewards. We suggest that this preference may be driven by the impact of the time course rather than the changes in reward magnitude alone. For example, when monetary options in the monetary choice task are ranked from lowest to highest with respect to their corresponding *k* coefficients, there is a 70% escalation from the lowest medium-sized reward (£50) to the highest large-sized reward (£85), whereas the delay escalation across these monetary choices is approximately 303% (from 30 to 91 days). This means that the reward value per unit of time dramatically decreases, and it is possible that patients with MDD are more sensitive to these changes. It has been argued that individuals with impairments in time perception may have an altered perception of distant reward magnitude based on a higher cost per time unit (Wittmann & Paulus, 2008). There is some evidence to suggest that patients with MDD may have distorted time perception, experiencing a slowing effect on time relative to healthy subjects and patients with bipolar disorder (Bschor *et al.* 2004). This could mean that patients with MDD perceive delays as longer than they really are, thus devaluing delayed rewards by associating a higher overall cost for delays even if their cost per unit of delay is comparable to healthy subjects.

Paradoxically, impairments evaluating rewards over time could enhance overall financial performance in the monetary choice task. For example, from an evolutionary financial point of view, steeper discounting behaviour in the monetary choice task, such as we observed in patients with MDD, can lead to individuals banking larger amounts of money at any given point in time. An alternative explanation of our findings is that patients with MDD may hold a more realistic view of their prospects at any time point. Depressive realism may be a mechanism by which patients with MDD hold a more accurate estimation of control over environmental contingencies and a more accurate evaluation of uncertainties between the present time and the future compared to healthy subjects (Moore & Fresco, 2012). Such realism may influence preferences for immediate rewards and confer an advantage in some specific contexts. However, the present task does not explicitly quantify such uncertainties about the future; the hypothesis could be tested explicitly by using an adaptation of the monetary choice task to test whether lowering the probability of receiving the delayed reward results in depressed patients outperforming controls.

Other studies have reported that patients with MDD may outperform healthy subjects in certain socially contingent decision-making paradigms, requiring sacrifice of financial rewards and investment of time to reach an optimal solution to a problem (Harle *et al.* 2010; von Helversen, 2011). Therefore, abnormalities in rewarded decision making in MDD may be advantageous in some contexts but disadvantageous in others, depending on specific task contingencies. This may have implications for occupational performance that warrant further exploration.

Our study had some limitations. First, the monetary choice task was hypothetical in nature. However, it has previously been shown that hypothetical monetary proposals produce discounting behaviour that is similar to that obtained in studies using real currency (Murphy *et al.* 2001). Second, in the present design, the reward magnitude and the delays were correlated, and therefore it is not possible to determine whether the effects we show here are driven by reward magnitude or reward delay; future studies could address this issue by having a design in which both factors vary independently. Third, although we have shown that temporal discounting for large-sized delayed rewards was particularly influenced by severity of hopelessness and overall impairments in psychosocial functioning, we did not use an external measure of impulsivity to rule out its possible confounding impact. However, we consider that impulsivity should have limited influence on delay discounting in MDD relative to addicted clinical populations. Finally, about half of our MDD group were currently medicated and therefore it is possible that some of the effects were driven by medication. A *post-hoc* comparison between medicated and unmedicated participants showed no significant difference; however, this issue should be explored in future studies with greater power to explore effects of medication (and other treatments).

## Conclusions

We have shown that patients with MDD have significantly higher discounting rates for future rewards relative to both healthy subjects and remitted patients whose discounting behaviour is comparable. Correlations between clinical measures and the discounting rates suggest that the differences between our groups are driven by depressive symptomatology, especially future directed pessimism. We have also shown that patients with MDD are less sensitive to changes in the reward size, as indicated by discounting rates that plateau from middle- to large-sized rewards. We suggest that the overall costs associated with long delays may be driving steeper devaluation of the magnitude of the reward.

## Supplementary Material

Supplementary Material Supplementary information supplied by authors.

## Supplementary material

For supplementary material accompanying this paper, please visit http://dx.doi.org/10.1017/S0033291713002584.
