# Supplementary material for: Temporal discounting in major depressive disorder
Source: Psychol Med. 2013 Nov 1;44(9):1825–34. doi: 10.1017/S0033291713002584 (PMC4035754; doi:10.1017/S0033291713002584)
Supplement: Supplementary Material — Supplementary information supplied by authors. [file S0033291713002584sup001.doc]

**Supplementary Table S1.** Clinical characteristics of current MDD group (N=24)

| ***MD subtype*** |  |
| --- | --- |
| With melancholic features | 20/24 |
| With atypical features | 1/24 |
| No specific subtype | 3/24 |
| ***Antidepressant medication and other forms of treatment at time of study*** |  |
| SSRI antidepressant (Fluoxetine, citalopram, sertraline) | 8/24 |
| SNRI antidepressant (Venlafaxine, duloxetine) | 2/24 |
| Melatonin receptor agonist (Agomelatine) | 1/24 |
| No medication | 13/24 |
| ***Co-morbidity at time of study*** |  |
| Binge eating disorder | 3/24 |
| Generalised anxiety disorder | 3/24 |
| Panic disorder | 4/24 |
| Social phobia | 6/24 |
| Agoraphobia without panic disorder | 3/24 |
| Specific phobia (Shark) | 1/24 |
| Dysthymic disorder | 4/24 |
| ***Life-time axis-I co-morbidity**** |  |
| Post-traumatic stress disorder | 6/24 |
| Panic disorder | 2/24 |
| ***Co-morbid disorders in partial remission*** |  |
| Panic disorder | 1/24 |
| Post-traumatic stress disorder | 1/24 |
|  |  |

* All co-morbid disorders were fully remitted at time of study, unless otherwise specified. None of the co-morbid disorders was a likely primary cause of the depressive episodes. SSRI=selective serotonin reuptake inhibitor, SNRI=serotonin norepinephrine reuptake inhibitor. MDD subtype classification was based on adapting the SCID-I for DSMIV-TR to allow lifetime assessment of the subtypes. All medication-free participants had stopped medication well before the required washout phase. Co-morbid disorders in partial remission indicate presence of subclinical threshold symptoms.

**Supplementary Table S2.** Clinical characteristics of rMDD group (N=29)

| ***MD subtype*** |  |
| --- | --- |
| With melancholic features | 20/29 |
| With psychotic features | 1/29 |
| No specific subtype | 8/29 |
| ***Number of previous MDEs*** |  |
| 1 | 9/29 |
| 2 | 7/29 |
| 3 | 7/29 |
| 4 ≤ | 6/29 |
| ***Last MDE details*** |  |
| Average length of MDE (months ± SD) | 4.2±3 (range: 0.5-12) |
| Average time in remission (months ± SD) | 57.1± 59.4 (range:3-192) |
| ***Life-time axis-I co-morbidity**** |  |
| Obsessive-compulsive disorder | 1/29 |
| Generalised anxiety disorder | 2/29 |
| Panic disorder | 4/29 |
| Social phobia | 1/29 |
| Generalised anxiety disorder (NOS) | 1/29 |
| Specific phobia (Insect) | 2/29 |
| No life-time co-morbidity | 18/29 |

* All co-morbid disorders were fully remitted at time of study. None of the co-morbid disorders was a likely primary cause of the depressive episodes. MDD subtype classification was based on adapting the SCID-I for DSMIV-TR to allow life-time assessment of the subtypes.
